# Ipsilateral Putamen and Insula Activation by Both Left and Right GB34 Acupuncture Stimulation: An fMRI Study on Healthy Participants

**DOI:** 10.1155/2016/4173185

**Published:** 2016-12-08

**Authors:** Sujung Yeo, Maurits van den Noort, Peggy Bosch, Sabina Lim

**Affiliations:** ^1^College of Korean Medicine, Sang Ji University, Wonju 26339, Republic of Korea; ^2^Department of Meridian & Acupoint, College of Korean Medicine, WHO Collaborating Center for Traditional Medicine, East-West Medical Research Institute, Kyung Hee University, Seoul 130-701, Republic of Korea; ^3^Donders Institute for Brain, Cognition and Behaviour, Radboud University Nijmegen, 6525 HR Nijmegen, Netherlands

## Abstract

The modulatory effects on the brain during right versus left side acupuncture stimulation of the same acupuncture point have been a subject of controversy. For clarification of this important methodological issue, the present study was designed to compare the blood oxygen level-dependent responses of acupuncture stimulation on the right versus left* Yanglingquan* (GB34). Twenty-two healthy subjects received right or left GB34 acupuncture. Our results show that acupuncture on the left GB34 induced neural responses in the left putamen, caudate body, insula, postcentral gyrus, claustrum, right and left thalamus, right middle frontal gyrus, hypothalamus, and subthalamic nucleus. Acupuncture on the right GB34 induced neural responses in the right middle frontal gyrus, inferior parietal lobule, thalamus, putamen, lateral globus pallidus, medial globus pallidus, and insula. Interestingly, the putamen and insula were ipsilaterally activated by acupuncture on either the left or right GB34; therefore, they seem to be the main target areas affected by GB34 acupuncture. This is the first reported functional magnetic resonance imaging study directly comparing needling on the right and left GB34. Although more replication studies are needed, our preliminary results prove that acupuncture has different modulatory effects on the brain when performed on the right versus left side.

## 1. Introduction

Acupuncture is an important modality of complementary medicine and is used in the treatment of pain, mood, and autonomic-related disorders [1–3]. In spite of its long history and public acceptance for medical management, the mechanisms of acupuncture are still unclear [4]. One leading investigatory approach includes mapping or localizing acupuncture-associated changes in the brain's functions [5]. Several tools have been applied to explore the central effects of acupuncture on the state of the brain. In particular, functional magnetic resonance imaging (fMRI) has been used extensively [6–8] because it can indirectly measure the brain's activity and the functional changes in the brain's state as well as discover connectivity patterns, in response to acupuncture without radiation or invasive procedures [9].

fMRI has been frequently applied for research on the neurophysiological mechanisms and the effects of acupuncture [10–13]. The majority of these studies used an experimental paradigm of acupuncture stimulations on only one side, that is, the left [14, 15] or the right acupuncture point [16, 17], but in some studies, both the left and the right side points were stimulated [10, 18]. Still, the neuronal effects of left side versus right side acupuncture at a particular acupuncture point are controversial. Hui and colleagues [10] suggested that a consistent modulation of multiple bilateral cortical and subcortical limbic and paralimbic structures was observed during acupuncture manipulations at LI4, no matter whether the right LI4 or the left LI4 was stimulated. However, not all results point in the same direction; for instance, bilateral deactivations of the brain were found during acupuncture stimulation at the left LI4 [14] while bilateral activations and deactivations were found during acupuncture stimulation at the right LI4 [19].

In this study, we will focus on the acupuncture point* Yanglingquan* (GB34) with respect to the right side versus the left side neuronal-effects controversy. GB34 has been used to treat motor dysfunctions such as hemiplegia, a variety of muscle disorders, knee pain [20], and Parkinson's disease (PD) [7, 21]. Particularly in a Parkinsonism animal model, acupuncture was found to prevent neuronal death [22, 23] and to inhibit the microglial activation and inflammatory events in the substantia nigra-striatum dopaminergic system [24].

Several fMRI studies demonstrated that acupuncture stimulation at GB34 produced robust patterns of activity within the central nervous system. In these studies, acupuncture stimulation increased neural responses in regions that were impaired as a result of Parkinson's disease [7, 25]. fMRI studies on healthy participants revealed that acupuncture at GB34 specifically activated the putamen, caudate body, claustrum, thalamus, and cerebellum [26, 27]. However, so far, different neural effects in response to GB34 acupuncture have been found, which can be explained by various factors, such as differences in the ages of the subjects, the kinds of patients [28, 29], the recruitment and intervention, and the sides used for GB34 acupuncture stimulation [26, 27].

Therefore, to clarify this important methodological issue, with possible widespread clinical implications (for instance, how to best treat patients with Parkinson's disease), we designed the present study to compare the blood oxygen level-dependent (BOLD) responses to acupuncture stimulation at the right versus the left GB34. Based on previous research, we hypothesized that acupuncture stimulations at the right and the left GB34 would activate different neural areas, but at the same time, we expected that, among the various activated areas, we would find common areas (the so-called “key GB34 regions”) that had been activated in response to both right and left GB34 acupuncture.

## 2. Materials and Methods

### 2.1. Participants

Twenty-two healthy volunteers participated in this study. Written informed consent procedures, according to the institutional guidelines of the Human Research Committee of Kyung Hee Medical Hospital (reference number: KMC IRB 0861-06), were followed. Informed written consent was obtained from all participants, and this study was conducted in accordance with the Declaration of Helsinki-Ethical Principles for Medical Research Involving Human Subjects (http://www.wma.net/en/30publications/10policies/b3/). The participants did not receive any payment for their participation. The subjects included 11 males and 11 females, and their average age was 43.9 (range: 25–66) years. All participants were without history of any chronic disease, allergy, and neurological or psychiatric disorder and were all right-handed as verified by the Edinburgh Handedness Inventory [30]; the average score of the participants (± standard deviation) within the left GB34 group was 95% (± 5.00%), and the average score of the participants within the right GB34 group was 100% (± 0%).

### 2.2. Acupuncture

Acupuncture was conducted on the right and left* Yanglingquan* (GB34) by an experienced traditional Chinese medicine doctor, and the World Health Organization Standard Acupuncture Point Locations were followed. GB34 is located on the fibular aspect of the leg in the depression anterior and distal to the head of the fibula (World Health Organization, 2008). For the acupuncture stimulations, stainless-steel needles (0.25 mm × 40 mm; Dong Bang Acupuncture, Inc., Seoul, Republic of Korea) were used. The needles were manually inserted into either the right GB34 (for the right GB34 group) or the left GB34 (for the left GB34 group) to a depth of approximately 1.0 cm. The following experimental paradigm was used for the acupuncture and the sham acupuncture stimulations. First, the needle remained at rest for 1 minute and was then subjected to 1 minute of bidirectional rotation at 1 Hz (i.e., one second to the left, one second to the right, etc., for a total of 1 minute). Then, the needle was at rest for 1 minute, followed by 1 minute of bidirectional rotation again, and finally, the needle was not moved for another 1 minute of rest. For sham acupuncture stimulation, a blunt needle was used [29] to generate a somatosensory response by gently contacting the skin at the right GB34 (for the right GB34 group) or at the left GB34 (for the left GB34 group) without needle insertion. All other aspects followed the same paradigm as for the previously described acupuncture condition.

### 2.3. Experimental Design and Procedure

The participants received general instructions for the experiment, followed by more detailed instructions for specific tasks. First, participants provided signed informed consent. Then, all participants completed the Edinburgh Handedness Inventory [30]. After that, the participants were reminded of the specific task instructions for the fMRI experiment and were instructed not to move their bodies and to be particularly careful not to move their heads during the imaging procedures. The scanning started with the sham acupuncture condition, which took about 5 minutes. Then, structural images were acquired for about 15 minutes. After the structural imaging, acupuncture was performed and lasted for about 5 minutes. Finally, after the sham acupuncture and the acupuncture scan blocks, the participants rated the intensities of the sensations they had felt [31]. At the end of the experiment, all the participants were debriefed. The total duration of the experiment was about 45 minutes.

### 2.4. MRI Data Acquisition

The data acquisition was conducted using a Philips 3.0 T MR scanner that was equipped for echo planar imaging (EPI). In total, 150 contiguous EPI functional volumes for the acupuncture and the sham acupuncture conditions (repetition time (TR) = 2000 ms, echo time (TE) = 35 ms, flip angle = 90°, slice thickness = 4.5 mm, number of slices = 30, matrix = 96 × 128, field of view (FOV) = 230 × 182 × 135 mm^3^, and acquisition voxel size = 2.4 × 2.4 × 4.5 mm^3^) were collected per individual. The participants were reminded of the instructions to rest with their eyes closed and not to move. During the scanning, participants wore earplugs to reduce discomfort due to MRI gradient noise. The participants remained comfortably in a supine position, and their heads were immobilized with support cushions in order to prevent movement. Finally, a high-resolution T1-weighted anatomical image was acquired for each participant by using a magnetization prepared gradient echo sequence (TR = 9.9 ms, TE = 4.6 ms, flip angle = 90°, slice thickness = 1 mm, number of slices = 196, matrix = 236 × 240, FOV = 235 × 235 × 196 mm^3^, and acquisition voxel size = 1 × 1 × 1 mm^3^) to allow spatial normalization and localization.

### 2.5. Behavioral Data Analysis

The Statistical Package for the Social Sciences (SPSS) version 22.0 (SPSS Inc., Chicago, UL, USA) was used for all statistical analyses. Average scores for age, cultural background, handedness, and* deqi* (i.e., the total* deqi* score, and 12 different* deqi* subscores: aching, dull pain, soreness, warmth, cold, tingling, throbbing, sharp pain, heaviness, numbness, fullness, and pressure) [31] were compared between the right GB34 group and the left GB34 group by using the Mann-Whitney *U*-test and the *t*-test. A significance level of *P* < 0.05 was used for all behavioral analyses.

### 2.6. Neuroimaging Data Analysis

The fMRI data were analyzed using SPM5 (http://www.fil.ion.ucl.ac.uk/spm/) (Welcome Department of Cognitive Neurology, London, UK). The first five volumes of each participant's dataset were discarded to allow for MR signal equilibration. The functional EPI-BOLD images were realigned, and the subject-mean functional MR images were coregistered with the corresponding structural MR images. These images were spatially normalized and transformed into a common space, following the definition of the SPM Montreal Neurological Institute (MNI) T1 template.

The fMRI data were then statistically analyzed using the general linear model and statistical parametric mapping of SPM5. At the first-level, single-subject fixed effect analyses were conducted. A model with the experimental conditions was tested in each participant's data separately. For second-level analysis, the generated contrast images for the main effects were assessed by conducting a one-sample *t*-test. The comparison between right GB34 group and the left GB34 group was assessed by conducting a two-sample *t*-test. Significant differences were accepted at a threshold of corrected cluster level *P* < 0.05. In addition, the cluster sizes and the peak *t* values of areas of significant change were determined. Relevant anatomical landmarks and Brodmann areas were identified by using GingerALE (http://www.brainmap.org/), which was developed at the Research Imaging Center of Texas, and were analyzed step by step. Moreover, all local maxima were reported as Talairach coordinates by using Talairach Client (http://www.talairach.org/). The voxels of the putamen, which had been activated by acupuncture stimulation at GB34, were measured using Rex [32]. Finally, the* deqi* scores and the MR signals of the putamen and the insula, which had been activated by acupuncture stimulation at GB34, were correlated using SPSS.

## 3. Results

### 3.1. Behavioral Results

The data for all 22 subjects were included in the behavioral analyses. All of the subjects reported* deqi* sensations. As can be seen in Table 1, the total* deqi* scores between the right GB34 group (mean ± standard error (SE): 16.82 ± 3.82) and the left GB34 group (mean ± SE: 13.00 ± 4.07) did not differ (*P* = 0.50).

### 3.2. Neuroimaging Results

#### 3.2.1. Areas of the Brain Activated by Acupuncture Stimulation on Either the Left or Right GB34

**Figure 1:**
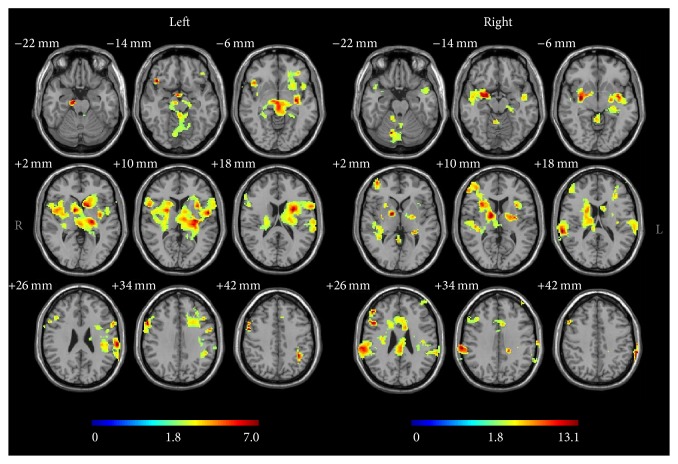
Brain activation during acupuncture stimulation on either the right or left GB34 (one-sample *t*-test with corrected cluster level *P* <  0.05). Bars show the *t* value.

**Figure 2:**
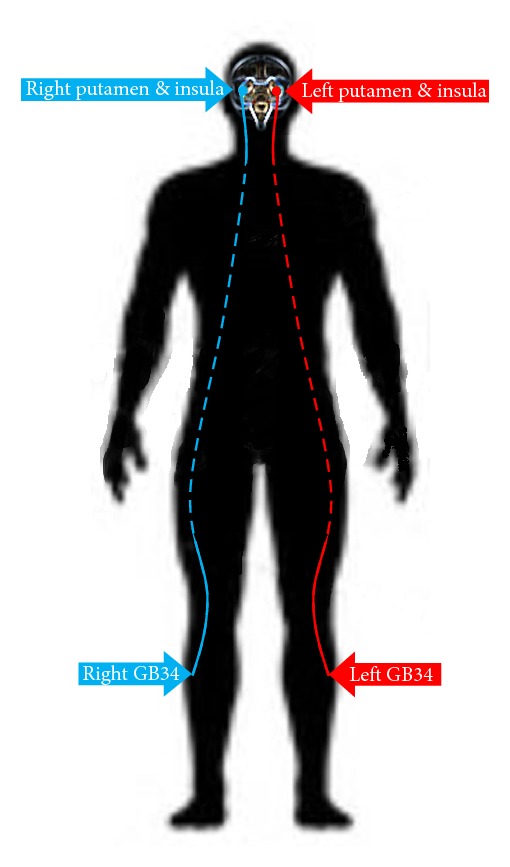
The putamen and insula were ipsilaterally activated by acupuncture stimulation at GB34.

Acupuncture stimulation on the left GB34 induced neural responses in the left putamen, left caudate body, left insula, left postcentral gyrus, left claustrum, right and left thalamus, right middle frontal gyrus, right hypothalamus, and right subthalamic nucleus (Figure 1 and Table 2). Acupuncture stimulation on the right GB34 induced neural responses in the right middle frontal gyrus, right inferior parietal lobule, right thalamus, right putamen, right lateral globus pallidus, right medial globus pallidus, right insula, right and left cerebellar tonsil, right and left culmen, and left uvula (Figure 1 and Table 2). Among these, the putamen and insula were ipsilaterally activated by acupuncture stimulation at GB34 (Figure 2).

#### 3.2.2. *Deqi* Sensation of Tingling Correlated with Activity in the Insula and Putamen Areas of the Brain

**Figure 3:**
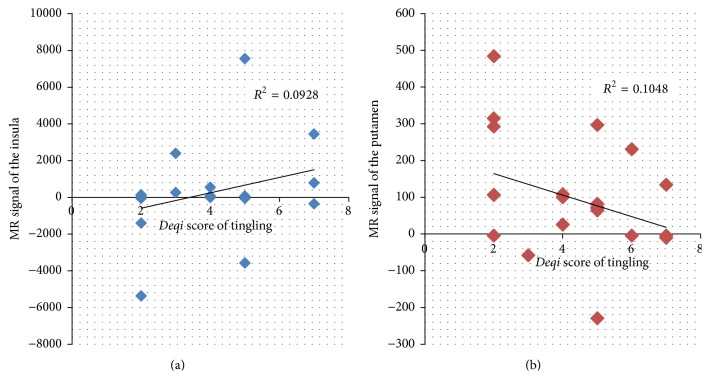
Variance in activity is accounted for by scores for the intensity of tingling during acupuncture. A positive correlation between the score for tingling and the mean MR signal change was shown in the (a) insula. A negative correlation between the score for tingling and the mean MR signal change was shown in the (b) putamen.

An increase in the intensity of the tingling sensation during acupuncture stimulation was associated with an increase in brain activity in the insula (Figure 3(a)). However, the tingling sensation showed a negative correlation with brain activity in the putamen (Figure 3(b)).

#### 3.2.3. Neural Response Comparison between Left and Right Acupuncture Stimulation at GB34

When the neural responses associated with left and right acupuncture were compared, those associated with left acupuncture showed stronger activation than those associated with right acupuncture (Table 3).

## 4. Discussion

The behavioral results of our study showed that all subjects reported* deqi* sensations. The total* deqi* score and the 12 different* deqi* subscores did not differ between the right GB34 group and the left GB34 group, indicating that the participants receiving right side GB34 acupuncture experienced the same feeling as those receiving left side GB34 acupuncture.

Our neuroimaging results revealed that acupuncture stimulation at the right and the left GB34 demonstrated a robust pattern of activation, with the putamen and the insula being activated in the ipsilateral hemisphere for both right and left side acupuncture stimulations; therefore, the putamen and the insula seem to be the so-called “key GB34 regions.” Note that the putamen is a part of the striatum, which is interconnected with many other structures, and that it works in conjunction with those other structures to control various types of motor skills [33], such as motor learning, motor performance and tasks [34], and motor preparation [35], and to specify the amplitudes of movement [36] and the movement sequences [33]. GB34 acupuncture has been reported to improve the performance of patients with Parkinson's disease on a finger tapping task [25]. Moreover, in animal research using a specific Parkinson's disease model, acupuncture at GB34 was found to prevent neuronal death [22, 23] in the putamen [24]. Taken together, our results seem to support the hypothesis that acupuncture stimulation at GB34 may control motor function by activation of the striatum. Acupuncture at GB34 was reported to be involved in motor function treatment [25], and the effect of treatment on the putamen is in line with previous findings in other motor task studies [37, 38]. More precisely, we found that GB34 acupuncture on healthy participants activated the putamen in the ipsilateral hemisphere. This finding is in line with the findings in previous studies, showing more dominant acupuncture-induced changes of BOLD signal intensities in the ipsilateral, as opposed to the contralateral, hemisphere. For instance, these ipsilateral acupuncture-induced changes were evident in the activation of the visual cortex induced by laser acupuncture at BL67 [39], as well as in the activation of the motor area induced by acupuncture manipulations at GB34 [27].

Besides the putamen, a robust pattern of activation in the insula in the ipsilateral hemisphere in response to right and left GB34 acupuncture was also found. The insula has been suggested to be involved in consciousness [40] and to play a role in diverse functions frequently linked to emotion [41] or the regulation of the body's homeostasis [42]. The insula is thought to have various functions, such as motor control, perception, self-awareness, and decision-making [43]. Acupuncture stimulation has been reported to activate the insula and to be involved in the regulation of the body's homeostasis [10, 44]. The present study demonstrates that acupuncture at either the right GB34 or the left GB34 activates the ipsilateral part of the insula. Moreover, an increase in the intensity of the tingling sensation during acupuncture stimulation was associated with an increase in brain activity in the insula (Figure 3(a)). This is in the line with previous research in which insular activation was found to be associated with* deqi* sensation [45] and in which the tingling sensation correlated positively with brain activity [46]. Our study also demonstrated that insular activation was positively associated with* deqi* sensation. A report in the literature [47] suggested that with deep stimulation at LV3, a gentle, repetitive manipulation producing mechanical pressure and tissue distortions activated more of the mechanoreceptors and the nociceptors that were innervated by thin myelinated A*δ*- and C-fibers. Previous electroencephalography (EEG) [48] and magnetoencephalography (MEG) [49] studies showed that selective stimulation of C-fibers induced ultra-late evoked brain potentials at the insula. Moreover, in a previous fMRI study, an increased activity in the insula due to C-fiber stimulation alone, compared to A*δ*-fiber stimulation alone [50], was found. In addition, a costimulation of C- and A-fiber inputs, as produced by usual large-area laser stimulations, was found to prevent the recording of ultra-late evoked brain potentials, potentials that could be recorded in response to selective stimulation of C-fibers [48, 50].

Interestingly, the tingling sensation showed a negative correlation with brain activity in the putamen. The negative association with tingling sensation may be the result of costimulation of C- and A*δ*-fiber inputs, leading to a repression of the central processing of the C-fiber input [46].

On the comparison of neural responses between left side and right side GB34 acupuncture, the neural responses for left side GB34 acupuncture showed stronger activations than those for right side GB34 acupuncture. This is in line with previous research reporting that the intensity of perceived pain was significantly higher for the left, as compared to the right hand [51]. Another study, which investigated the central effect of acupuncture on the left and the right LI4, showed a dissymmetry, indicating right hemisphere laterality [52]. However, we should mention that those results might have been affected by brain function lateralization [53–55]. Note that brain function lateralization is evident in the phenomena of right- or left-handedness [56], showing, for instance, that 95% of right-handed people have left-hemisphere dominance for language while only 70% of left-handed people have left-hemisphere dominance for language [57]. This tendency could affect information processing subject to needling. In the present study, all participants were right-handed because of our attempt at controlling this important methodological issue. Future studies are needed to further investigate this brain function lateralization and handedness issue in response to acupuncture stimulation; however, our first results stress the importance of carefully controlling this in fundamental and clinical acupuncture research.

Finally, our study has several limitations, which should be taken into account in order to correctly interpret its results. Note that the present study is the first reported fMRI study directly comparing needling at the right and the left side GB34; moreover, the present study included only 22 participants. Therefore, replication studies, if possible with larger samples, are required in order to verify the present findings. Although acupuncture at the right and the left GB34 was shown to have different effects, this study was conducted on healthy participants; therefore, replication studies are needed with right versus left GB34 acupuncture on patients with motor problems (e.g., patients with Parkinson's disease) [7]. Also, replication studies with different scanning procedures (e.g., the resting state fMRI acupuncture procedure [58] instead of the present block design) or with different neuroimaging techniques, such as EEG [59] or MEG [60], are warranted. Moreover, if the question of whether the difference between the effects of right side versus left side acupuncture stimulation is a general issue that needs to be considered in acupuncture treatment is to be answered, replication studies with acupuncture points other than GB34, for instance, with* Hegu* (LI4),* Zusanli* (ST36), and* Taichong* (LV3), are needed.

## 5. Conclusion

To the best of our knowledge, this is the first reported fMRI study directly comparing needling at the right and the left GB34. Although more replication studies are needed, our preliminary results showed that the putamen and the insula were key GB34 regions; moreover, proof was found for acupuncture's having different modulatory effects on the brain when performed on the right versus the left side, leading to important methodological and clinical implications. The brain function lateralization and handedness issue should be taken into account in designing future fundamental and clinical acupuncture studies, for instance, by carefully controlling handedness, and this might eventually result in more optimal individual (GB34) acupuncture treatments.

## Figures and Tables

**Table 1:** Sample characteristics: specified for the left versus the right GB34 acupuncture group.

Group	Left (*n* = 10)	Right (*n* = 12)	*P* value
Age	54.9 ± 2.43	56.58 ± 2.96	0.39
Gender	5 males/5 females	6 males/6 females	
Asian (%)	100%	100%	
Edinburgh Handedness Inventory	Right 95 ± 5.00%	Right 100 ± 0%	
Total deqi score	13.00 ± 4.07	16.82 ± 3.82	0.50
Aching score	Median 0, range 0~4	Median 0, range 0~5	0.39
Dull pain score	Median 0, range 0~3	Median 0, range 0~5	0.76
Soreness score	Median 0, range 0~4	Median 0, range 0~5	0.56
Warm score	Median 0, range 0~5	Median 0, range 0~4	0.56
Cold score	Median 0, range 0	Median 0, range 0~2	0.51
Tingling score	Median 4.5, range 0~6	Median 5, range 2~7	0.86
Throbbing score	Median 0, range 0~5	Median 0, range 0~4	0.86
Sharp pain score	Median 0, range 0~7	Median 1, range 0~7	0.35
Heaviness score	Median 0, range 0~7	Median 0, range 0~5	0.47
Numbness score	Median 0, range 0~5	Median 0, range 0~4	0.70
Fullness	Median 0, range 0~7	Median 0, range 0~4	0.56
Deep pressure score	Median 0, range 0~4	Median 0, range 0~5	0.35

Data are mean ± SEM or median, range.

**Table 2:** Areas of brain activation induced by acupuncture stimulation on either the left or the right GB34.

Regions	Left acupuncture					Right acupuncture				
	Talairach					Talairach				
	*X*	*Y*	*Z*	*t* value	BA^a^	*X*	*Y*	*Z*	*t* value	BA^a^
*Frontal lobe*										
Middle frontal gyrus R^b^	44.82	2.14	39.47	4.82	6	30.43	41.09	6.92	4.98	9, 10, 46
*Parietal lobe*										
Postcentral gyrus L^c^	−56.87	−29.48	20.34	4.45	40					
Inferior parietal lobule R						55.95	−32.45	27.37	4.02	40
*Sublobar*										
Thalamus R	8.21	−1.99	6.03	4.62		10.03	−5.9	7.49	5.95	
Thalamus L	−17.77	−18.8	5.8	5.01						
Putamen R						22.95	6.55	14.29	3.98	
Putamen L	−25.06	−1.47	1.91	7.03						
Lateral globus pallidus R						24.92	−9.01	0.24	6.62	
Medial globus pallidus R						15.7	−5.06	−1.34	4.09	
Caudate body L	−10.3	5.39	8.22	4.58						
Hypothalamus R	2.81	−6.33	−7.09	4.67						
Insula R						52.34	−31.74	20.17	5.57	13
Insula L	−43.7	−7.83	10	6.31	13					
Extranuclear R	32.52	14.36	−8.23	5.41	13					
Claustrum L	−25.27	−19.46	12.82	4.86						
*Midbrain*										
Subthalamic nucleus R	8.32	−13.99	−5.92	5.03						
*Cerebellum*										
Cerebellar tonsil R						34.44	−49.12	−32.22	13.14	
Cerebellar tonsil L						−4.44	−54.51	−33.39	3.82	
Culmen R						36.25	−45.75	−28.27	9.12	
Culmen L						0.82	−42.07	−6.9	4.05	
Uvula L						−11.92	−66	−31	3.71	

^a^Brodmann area, ^b^R = right hemisphere, and ^c^L = left hemisphere.

**Table 3:** Neural response comparison between left and right GB34 acupuncture stimulation.

Regions	Left > right					Left < right				
	Talairach					Talairach				
	*X*	*Y*	*Z*	*t* value	BA^a^	*X*	*Y*	*Z*	*t* value	BA^a^
*Frontal lobe*										
Middle frontal gyrus R^b^						32.4	40.91	8.7	4.42	10
Medial frontal gyrus L^c^	−10.83	8.83	49.97	3.4	6					
Precentral gyrus L	−45.84	−4.5	33.7	3.3	6					
*Parietal lobe*										
Postcentral gyrus L	−29.43	−37.48	43.47	3.51	3					
*Sublobar*										
Thalamus L	−23.45	−23.37	14.28	3.24						
Putamen L	−25.32	−3.38	21.55	5.29						
Caudate body L	−8.43	5.55	6.46	3.91						
Hypothalamus R	2.81	−4.47	−6.91	3.9						
Insula L	−43.67	−7.66	8.22	5.26	13					
*Limbic lobe*										
Parahippocampal gyrus R	15.58	−40.63	−2.91	3.32	30					
Cingulate gyrus L	−14.59	−4.37	50.46	3.26	24					
*Midbrain*										
Subthalamic nucleus R	8.29	−16.02	−4.31	3.71						
*Cerebellum*										
Cerebellar tonsil R	19.63	−60.05	−35.31	3.4						
Fastigium L	−8.29	−57.4	−22.92	3.24						

^a^Brodmann area, ^b^R = right hemisphere, and ^c^L = left hemisphere.
